# BRD4 Inhibition Attenuates Inflammatory Pain by Ameliorating NLRP3 Inflammasome-Induced Pyroptosis

**DOI:** 10.3389/fimmu.2022.837977

**Published:** 2022-01-26

**Authors:** Tong Hua, Haowei Wang, Xiaoyi Fan, Ni An, Jian Li, Honghao Song, Erliang Kong, Yongchang Li, Hongbin Yuan

**Affiliations:** ^1^ Department of Anesthesiology, Changzheng Hospital, Naval Medical University, Shanghai, China; ^2^ National Medical Products Administration (NMPA) Key Laboratory for Research and Evaluation of Narcotic and Psychotropic Drugs, Xuzhou, China; ^3^ Chinese People’s Liberation Army, Liao Yang, China

**Keywords:** inflammatory pain, BRD4, neuron, NLRP3 inflammasome, pyroptosis

## Abstract

Chronic pain, such as persistent inflammatory pain, remains a public health problem that has no effective treatment at present. Bromodomain-containing protein 4 (BRD4) inhibition, induced by JQ1 injection or BRD4 knockdown, has been used to attenuate inflammatory pain; However, it remains elusive whether BRD4 aggravates inflammatory pain by regulating inflammasome. Western blot and immunofluorescence staining showed that BRD4 expression increased after administration of complete Freund’s adjuvant (CFA) and reached its peak on day 3. Immunofluorescence staining showed that BRD4 was mainly colocalized with NeuN-positive neurons in the spinal cord, which was accompanied by upregulation of inflammasome component proteins, such as NLRP3, gasdermin D, and caspase-1. JQ1 was intrathecally injected into mice 1 h before CFA administration, and the mechanical and thermal hyperalgesia levels were measured on days 1, 3, and 7 after CFA administration. CFA-induced inflammatory pain, paw inflammation, and swelling were attenuated by pre-treatment with JQ1. To our knowledge, this study was the first to prove that NLRP3 inflammasome-induced neuronal pyroptosis participates in inflammatory pain. BRD4 inhibition decreased the expression of pyroptosis-related proteins by inhibiting the activation of NF-κB signaling pathway, both *in vivo* and *in vitro*. Taken together, BRD4 inhibition exerted analgesic and anti-inflammatory effects against inflammatory pain by inhibiting NF-κB and inflammasome activation, which protected neural cells from pyroptosis.

## 1 Introduction

Pain plays an essential role in protecting human beings from damaging stimuli. However, continuing and serious peripheral tissue or nerve injury easily develops into chronic pain, such as inflammatory pain. Inflammatory pain is a common health issue that causes depression and may increase suicide occurrences. One fifth of the European population is affected by chronic pain, and most of the afflicted are women and elderly people (1). In addition, chronic pain causes a huge economic burden. More than €200 billion in Europe and $150 billion in the United States have been spent on chronic pain (2); however, chronic pain still lacks effective treatments. The drugs most commonly used to alleviate chronic pain are opioids and non-steroidal anti-inflammatory drugs (NSAIDs) (3). Long-term administration of opioids increases the incidence of serious adverse effects, such as drug addiction, opioid tolerance, and opioid-induced hyperalgesia (4); gastrointestinal adverse effects occur after long-term use of NSAIDs. Thus, more effective strategies against chronic pain are needed.

As a member of the Bromo and Extra-Terminal (BET) family, bromodomain-containing protein 4 (BRD4) contains two N-terminal bromodomains. BRD4 binds to the acetylated histones and transcription factors through N-terminal bromodomains and modulates many pathophysiological activities, including inflammation (5). BRD4 plays a vital role in aggravating acute gouty arthritis by regulating NF-κB/NLRP3/gasdermin D (GSDMD) signaling pathway (6). In addition, BRD4 accelerates colonic injury during endotoxemia, a serious inflammation response related to infection (7). Complete Freund’s adjuvant (CFA) -induced inflammatory pain increases the expression of BRD4, ultimately resulting in enhanced excitability of nociceptive neurons and thermal hyperalgesia (8). As one of the most commonly used BRD4 inhibitors, JQ1 attenuates the recruitment of BRD4 to the promoters of genes related to inflammation and cancer (9). Spinal cord injury usually causes severe neuroinflammation, but JQ1 promotes the spinal cord repair by reducing pro-inflammatory mediators and increasing the expression of anti-inflammatory cytokines (10, 11). Inhibition of BRD4 is a promising way to alleviate CFA-induced inflammatory pain and neuroinflammation.

Pyroptosis, induced by canonical and non-canonical inflammasomes, is a type of programmed cell death discovered recently. Canonical inflammasomes, such as the NLRP3 inflammasome, activate caspase-1, whereas non-canonical inflammasomes activate mouse caspase-11 or human caspase-4 (12). GSDMD, a member of the gasdermin protein family, is then cleaved by inflammatory caspases induced by inflammasome activation, becoming the N-terminal domain form (GSDMD-N). GSDMD-N binds with acidic phospholipids to form pores that disrupt plasma membrane integrity, increasing the release of mature interleukin (IL)-1β and mature IL-18, which are processed by cleaved-caspase-1 (13). As a member of the canonical inflammasomes, the NLRP3 inflammasome is widely studied in inflammatory pain (14, 15). Electroacupuncture treatment relieves inflammatory pain by inhibiting the NLRP3 inflammasome (14). However, the role of the NLRP3 inflammasome in inflammatory pain is controversial. In acute inflammatory pain induced by carrageenan, the NLRC4 inflammasome, not NLRP3, participates in the genesis of hyperalgesia (16). This process might involve a different inflammatory pain model. Despite these important studies related to the NLRP3 inflammasome, the potential mechanism of GSDMD-induced pyroptosis in inflammatory pain remains unknown. BRD4 aggravates many pathophysiological activities through GSDMD-induced pyroptosis (7, 17). However, the relationship between BRD4 and pyroptosis in inflammatory pain remains elusive.

In this study, we found that GSDMD-induced neuronal pyroptosis participated in the genesis of mechanical and thermal hyperalgesia. The BRD4 inhibitor JQ1 or BRD4 knockdown attenuated CFA-induced inflammatory pain by reducing neuronal pyroptosis.

## 2 Methods

### 2.1 Experimental Animals

Wild-type C57BL/6J male mice (8 weeks old) were provided by the Animal Experiments Center of Naval Medical University (NMU) (Shanghai, China). All mice were housed in standard cages in a condition of 23°C and 55% humidity under a 12 h-12 h light-dark cycle. All animal experiments were approved by the Scientific Investigation Committee of NMU, and the guidelines of the Ethics Committee of the International Pain Research Association were followed.

### 2.2 CFA-Induced Inflammatory Pain Model and Drug Administration

Inflammatory pain was induced by CFA (F5881, Sigma-Aldrich), as previously described (18). Briefly, mice were anesthetized with 1% Pentobarbital sodium (50 mg/kg, injected intraperitoneally); 20 µL of CFA or saline was intraplantarly injected into the left hind paw from heel to sole. The mice were divided into the following groups: sham (saline), control (CFA), and JQ1(CFA+JQ1) (MCE, Shanghai, China). Mice in the CFA+JQ1 group mice were intrathecally injected with 100 mM (5µL) of JQ1 1 h before CFA injection, as previously described (8). The sham and control groups were intrathecally injected with 5µL of saline.

### 2.3 Cell Culture and Intervention Measures

HT22, a mouse hippocampal neuron cell line, was purchased from American Type Culture Collection. HT22 cells were cultured in DMEM (L110KJ, Basal Media, China) containing 10% fetal bovine serum (FBS, 10099141, Gibco), 100 U/mL of penicillin, and 100 µg/mL of streptomycin (15070063, Gibco) and grown in a condition of 5% CO2 and 95% humidity at 37°C. HT22 cells were treated with different doses of JQ1 for 24 h, and a drug toxicity test using a Cell Counting Kit 8 (CCK8) was used to find the optimal dose. Then, after pre-treatment with 100 nM or 500 nM of JQ1 for 1 h, HT22 cells were sequentially stimulated with LPS (1 µg/mL) for 24 h and ATP (5 mM) for 30 min to mimic cell pyroptosis.

### 2.4 Behavioral Tests

#### 2.4.1 Mechanical Hyperalgesia

Mice were adapted to the room environment in a brown Plexiglas box for 30min on a wire mesh platform before their paw mechanical withdrawal thresholds (PWT) were evaluated using the electronic von Frey test (IITC/Life Science, CA, USA) with an appropriate probe that stimulated the hind paw plantar with incremental forces. When a force induced a paw withdrawal response, the mechanical pressure that evoked the response was recorded through the pressure transducer in the probe. The tests were duplicated three times with 5-min stimulation intervals, and the average value of the three duplicated tests was recorded as the PWT. The baseline PWT was obtained 1 day before the inflammatory pain model was constructed, and mechanical hyperalgesia was tested on days 1, 3, and 7 after the CFA or saline administration and on days 1, 3, and 7 after intrathecal injection with JQ1.

#### 2.4.2 Thermal Hyperalgesia

Mice were adapted to the room environment in a transparent plastic box for 30min on a glass surface before their thermal withdrawal latency (TWL) was evaluated using a Hargreaves radiant heat apparatus (IITC/Life Science, CA, USA) that stimulates the hind paw until paw-withdrawal response was induced. The response was shown as foot lifting, dodging, or flinching. A cutoff of 20 s was set to avoid tissue damage. The baseline TWL was obtained 1 day before CFA or saline administration. Thermal hyperalgesia was tested on days 1, 3, and 7 after the CFA or saline administration and on days 1, 3, and 7 after intrathecal injection with JQ1. Three measurements were obtained, with 5-min intervals for each test, and the average was taken as the TWL.

### 2.5 Cell Viability CCK-8 Assay

To detect the effect of JQ1 treatment on the viability of HT22 cells, a CCK-8 kit (MCE, Shanghai, China) was used. Briefly, 1×10^3^ HT22 cells were seeded in each well of a 96-well plate and treated with different concentration of JQ1 (10, 50, 100, 500, and 1000 nM) for 24 h. 100 μL of complete medium mixed with 10 μL of CCK-8 reagent was added to each well for 1 h. Finally, the absorbance was detected using the infinite M200 PRO (TECAN) at 450 nm.

### 2.6 Western Blot

The L4-L5 spinal cord tissue of modeled mice or treated HT22 cells was homogenized in RIPA Lysis Buffer (Epizyme, Shanghai, China) containing a protease and phosphate inhibitor (Epizyme). The homogenates were centrifuged at 12,000 × *g* for 10 min. Then, the supernatant liquid was collected. BCA protein assay kit (Epizyme) was used for testing protein concentrations. Equal amounts of proteins were separated on a 10% SDS PAGE and were wet-electro-transferred onto a 0.2 μm polyvinylidene fluoride (PVDF) membranes (Millipore, USA). The membrane was blocked with Protein Free Rapid Blocking Buffer (Epizyme) for 1 h at room temperature on a rocker and then was incubated with specific primary antibody overnight at 4°C. The primary antibodies used were anti-GAPDH (1:1000 dilution, 5174), anti-NLRP3 (1:1000 dilution, D4D8T), NF-κB-p65 (1:1000 dilution, 93H1), anti-GSDMD-F (1:1000 dilution, E9S1X), anti-GSDMD-N (1:1000 dilution, E9S1X), and anti-mature IL-1β (1:1000 dilution, 63124), all purchased from cell signaling technology; anti-BRD4 (1:1000 dilution, servicebio, GB111415); anti-caspase-1-p20 (1:1000 dilution, Santa cruz, sc-398715). The following day, the membrane was washed with TBST (Servicebio, Wuhan, China) three times for 10 min each time, and then incubated with anti-rabbit secondary antibody (1:10,000 dilution, proteintech, SA00001-2) or anti-mouse secondary antibody (1:10,000 dilution, proteintech, SA00001-1) at room temperature for 2 h. Proteins were measured by enhanced chemiluminescence (ECL) reagent (Simuwu, China), imaged with a gel imaging system (Tanon, China), and quantified using ImageJ software (NIH, Bethesda, MD, USA).

### 2.7 Hematoxylin and Eosin Staining

Left paw tissues were fixed in buffered 4% paraformaldehyde for 24 h. Then, paw tissues were washed with running water for 3-5 h. Tissues were then routinely dehydrated by alcohol, transparentized, waxed, and embedded with paraffin. Paraffin blocks were cut into thin sections with a thickness of 5 µm. The sections were deparaffinized, hydrated, and stained with hematoxylin and eosin (H&E) solution according to a previously described protocol (7). Images were acquired using light microscopy (Olympus, Tokyo, Japan).

### 2.8 Quantitative Real-Time Polymerase Chain Reaction

The total RNA of the tissues or cells was extracted using TRIzol (R401-01, Vezyme). The RNA was reverse-transcribed to cDNA with a reverse transcription kit (11141EZ60, YESEN) according to the manufacturer’s instructions. An SYBR Green kit (11202ES03, YESEN) was used for polymerase chain reaction (PCR) quantification on the QuantStudio 5 (Applied Biosystems). The cycle threshold (Ct) values of target proteins were collected and normalized to that of β-actin, and the fold change of gene expression was calculated with the 2^-ΔΔCT^ method. The primers sequence used are as follows: β-actin Forward: 5′-GGCTGTATTCCCCTCCATCG-3′, β-actin Reverse: 5′-CCAGTTGGTAACAATGCCATGT-3′; BRD4 Forward: 5′-GTGAGAAGCTAGGCCGTGTAG-3′, BRD4 Reverse: 5′-AGGCAGGACCTGTTTCAGAGT-3′.

### 2.9 Immunofluorescence

After deep anesthesia with an overdose of pentobarbital sodium, mice were intracardially perfused with PBS until the effluent was clear and bloodless; then, they were injected with 4% ice-cold paraformaldehyde (PFA) until their limbs became stiff. The L4-L5 spinal cord tissues were collected and fixed in PFA for 5 h, and then dehydrated using a 25% sucrose solution until the tissues sunk to the bottom of the tube. The tissues were embedded in O.T.C tissue freezing medium (SAKURA, USA) and frozen to −20°C in a cryostat (Leica, Germany). Each sample was sectioned into 20-μm-thick slices, as previously described (19). A single or double labeling method was used for immunofluorescence. Single-labeling involved incubation of slices from target sections with the BRD4 antibody (Abcam, ab128874) at room temperature overnight, and then with the Cy3-conjugated anti-rabbit IgG (Servicebio, GB21403) for 1 h. Slices were washed with TBST three times (10 min each time) after every incubation. Observation and photographic documentation were conducted using a fluorescence microscope (EclipseE600, Nikon, Japan). To explore the cell types that expressed BRD4 and pyroptosis-related molecules, the sections were incubated with primary antibodies against Iba1 (Servicebio, GB11105), GFAP (Servicebio, GB11096) or NeuN (Servicebio, GB11138) together with caspase-1-p20 (Santa Cruz, sc-398715) or GSDMD (Santa Cruz, sc-393656), respectively. Then, the sections were incubated with secondary antibodies at room temperature for 1 h, washed again. Slices were imaged with a fluorescence microscope.

### 2.10 Small-Interfering RNA and Transfection

BRD4 small-interfering RNA (siRNA, Ribo, Shanghai) and a negative control (si-NC) were used to transfect HT22 cells at a confluency of 70-90% using Lipofectamine 3000 (Life Technologies) according to the manufacturer’s instruction. After 48 h, HT22 cells were sequentially stimulated with LPS (1 μg/mL) for 24 h and ATP (5 mM) for 30 min. The gene sequences of the negative control and the BRD4 siRNA used were GCTCAAGACACTATGGAAA and GGTACCAAACACAACTCAA, respectively.

### 2.11 Data Analysis

Data were expressed as the mean ± standard error of the mean (SEM), and results were analyzed using GraphPad Prism 7 software. A two-tailed Student’s t-test was used for statistical analysis of two groups, and one-way analysis of variance was used for multiple comparisons with Bonferroni *post hoc* analysis. Differences were considered statistically significant if the *P* value was <0.05.

## 3 Results

### 3.1 BRD4 Expression Increased During CFA-Induced Inflammatory Pain

**Figure 1:**
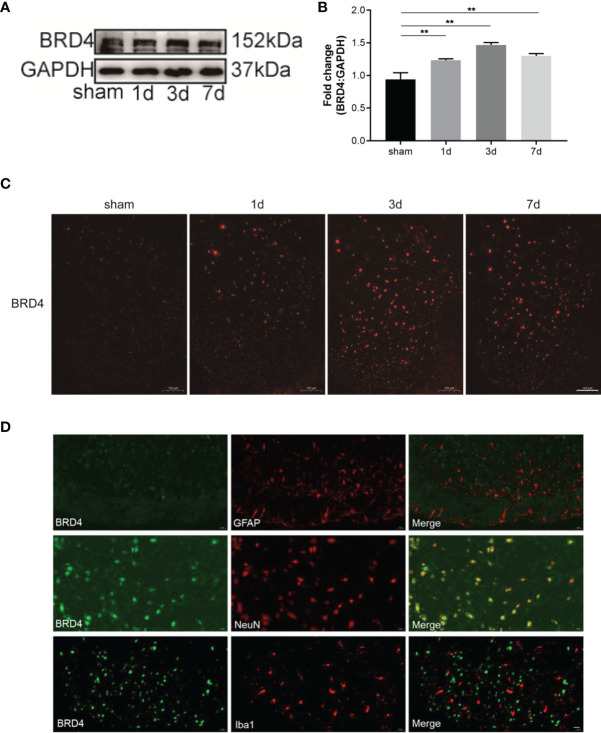
BRD4 expression is increased during complete Freund’s adjuvant (CFA)-induced inflammatory pain. **(A)** BRD4 expression levels in the dorsal horn of the spinal cords were tested by using western blot on days 1, 3, and 7. **(B)** Subsequent quantitative analysis of BRD4 expression. **(C)** BRD4 expression was also reflected by Immunofluorescence intensity in the dorsal horn of the spinal cord at different groups. Bar=100 µm **(D)**. Double immunofluorescence staining of BRD4 with microglia marker iba1 or astrocyte marker GFAP, neuron marker NeuN. Bar=20 µm. Data were presented as mean ± standard deviation (SD), n=4 mice/group. (**P < 0.01).

To analyze the role of BRD4 in inflammatory pain, the CFA paw injection model was used to simulate chronic inflammatory pain caused by peripheral tissue injury. The expression of BRD4 in the spinal dorsal horn was detected before and after CFA injection. The expression level of BRD4 protein in the spinal cord was significantly increased and peaked on the third day after CFA injection (**Figures 1A, B**), whereas the expression level was not significantly changed in the sham group. Consistent with western blot results, the mean fluorescence intensity of BRD4 in immunofluorescence staining was significantly enhanced by CFA injection (**Figure 1C**). The mRNA level of BRD4 was measured by qualitative PCR and was remarkably up-regulated, reaching a peak on the seventh day after CFA injection (**Figure S1**). The cellular localization of BRD4 in the spinal cord was also explored by immunofluorescence. BRD4 was mainly colocalized with NeuN-positive neurons in the spinal cord (**Figure 1D**), rather than with Iba1 (microglia maker) or GFAP (astroglia marker) positive cells. In summary, these results showed that the BRD4 in the spinal dorsal horn was mainly expressed in neurons and was elevated after inflammatory pain.

### 3.2 CFA-Induced Inflammatory Pain and Paw Inflammation Was Attenuated by JQ1, the BRD4 Inhibitor

**Figure 2:**
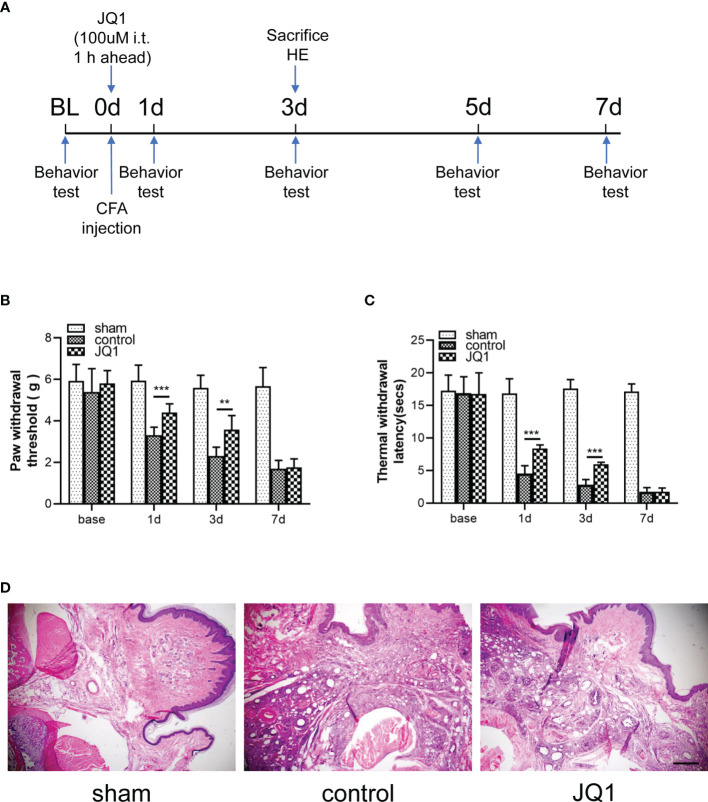
CFA-induced inflammatory pain and paw inflammation was attenuated by JQ1, the BRD4 inhibitor. **(A)** Experimental design to test the antinociceptive effects of BRD4 inhibitor JQ1 in the CFA-induced inflammatory pain model. **(B, C)** Effects of BRD4 inhibitor JQ1 on thresholds of mechanical allodynia **(B)** and thermal hyperalgesia **(C)**. Tests were carried out before CFA injection and on days 1, 3,and 7, n=6 mice/group. **(D)** Inflammation in mouse paw tissues by H&E staining, n=3 mice/group, all figures were magnified by 400×. Data were presented as mean ± standard deviation (SD). (**P < 0.01, ***P < 0.001).

To evaluate the effects of JQ1 on CFA-induced inflammatory pain, the electronic von Frey test was performed before and after CFA, saline, or JQ1 injection. At 1 h before CFA injection, 100 μM (5 μL) of JQ1 was intrathecally injected, as previously described (8) (**Figure 2A**). CFA was injected subcutaneously into the plantar area of the left paw of mice. This inflammatory pain model could induce a persistent mechanical and thermal allodynia in the ipsilateral paw from 6 h to 14 days after CFA injection (20). Our experimental data were consistent with previous reports. Compared with the hind paw of sham mice, the paw withdrawal threshold in the control mice was decreased from day 1 to day 7 and reached the minimum value on day 7 (**Figures 2B, C**). The mechanical and thermal allodynia induced by CFA was predominantly reversed through JQ1 pre-treatment, suggesting that BRD4 played a vital role in inflammatory pain. CFA induced a severe inflammatory reaction and tissue swelling of the injected paw on the third day after injection (21). Similarly, the left paw swelling had an obvious difference with that of the sham group mice, manifesting as increased accumulation of infiltrated cells in H&E staining. The paw swelling was gradually resolved in the CFA+JQ1 group with that of the control group, manifesting as less inflammatory cell infiltration on day 3 (**Figure 2D**). In summary, JQ1 pre-treatment attenuated CFA-induced inflammatory pain and paw inflammation.

### 3.3 CFA-Induced Inflammatory Pain Induced Neuronal Pyroptosis by Activating NF-κB

**Figure 3:**
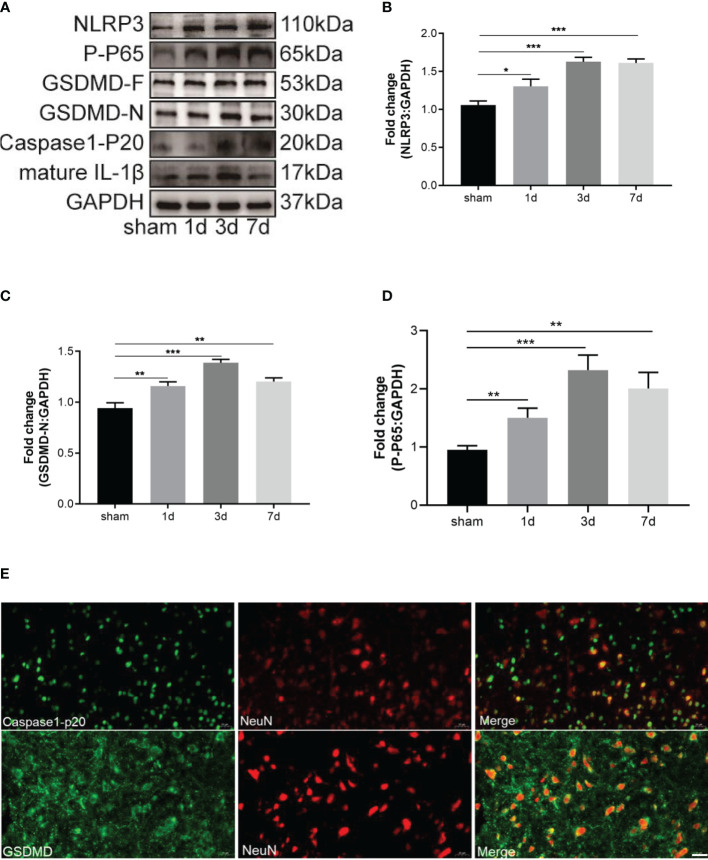
CFA-induced inflammatory pain induced neuronal pyroptosis through activating NF-κB. **(A)** Western blots of pyroptosis-related proteins, including NLRP3, GSDMD-F and GSDMD-N, caspase-1-P20, mature IL-1β. The activation of NF-κB signal transduction was tested by western blot of P-P65. **(B–D)** Subsequent quantitative analysis of NLRP3, GSDMD-N, P-P65 expression, n=4 mice/group. **(E)** Double immunofluorescence staining of GSDMD and caspase-1-P20 with neuron marker NeuN, Bar=20 µm, n=4 mice/group. Data were presented as mean ± standard deviation (SD). (*P < 0.05, **P < 0.01, ***P < 0.001).

The NLRP3 inflammasome promotes the beginning of cell pyroptosis. One study has shown that CFA-induced inflammatory pain increased the level of the NLRP3 inflammasome in the spinal dorsal horn (15). No study has measured the pyroptosis-associated proteins during inflammatory pain. The levels of NLRP3 inflammasome-related proteins (NLRP3, caspase-1-p20) were increased from day 1 to day 7 and reached a peak on day 3 (**Figures 3A, B**). GSDMD-F, GSDMD-N, and mature IL-1β which performs the function of pyroptosis-associated cells were detected by western blot analysis on days 1, 3, and 7 after CFA injection. Notably, compared with the sham group, the control group exhibited expressions of GSDMD-F, GSDMD-N, and mature IL-1β that were remarkably upregulated on days 1, 3, and 7 and that peaked on day 3 after CFA injection (**Figures 3A, C**). The NF-κB signaling pathway usually activates transcription of the NLRP3 inflammasome. Therefore, p65 phosphorylation was analyzed by western blot, which showed that it was up-regulated during inflammatory pain (**Figures 3A, D**). To uncover the main pyroptotic cell type, double labeling immunofluorescence staining was used. Results showed that caspase-1-p20 and GSDMD were mainly expressed in neurons (**Figure 3E**). Our data suggest that NLRP3 inflammasome-mediated pyroptosis played a vital role in CFA-induced inflammatory pain by activating NF-κB.

### 3.4 BRD4 Inhibitor Alleviated CFA-Induced Inflammatory Pain by Inhibiting Neuronal Pyroptosis

**Figure 4:**
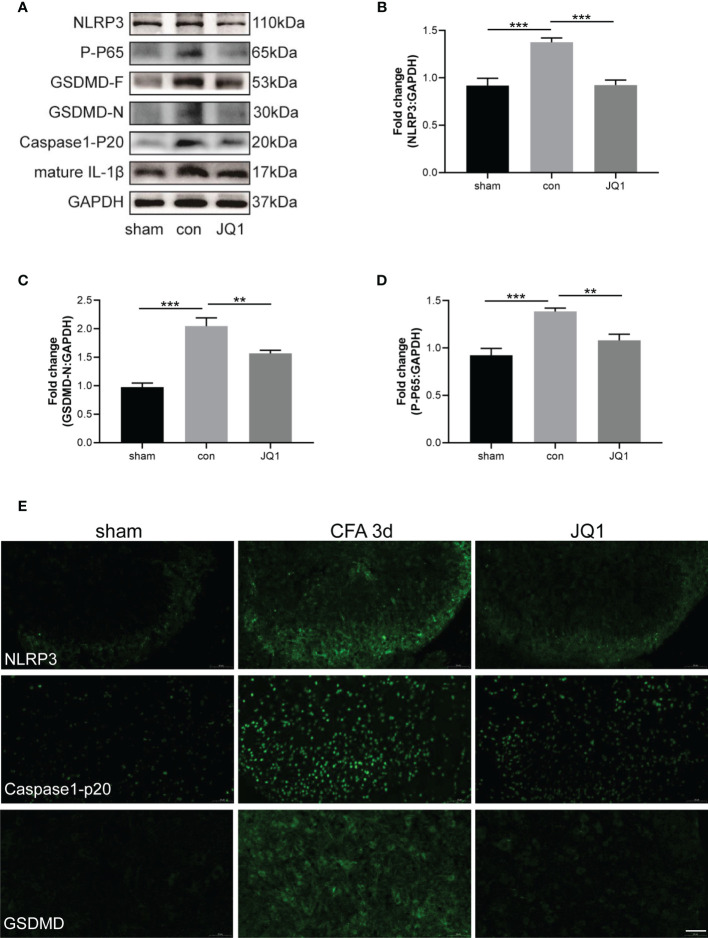
BRD4 inhibitor alleviated CFA-induced inflammatory pain by inhibiting neuronal pyroptosis. **(A)** Western blot assaied of NLRP3, GSDMD-F, GSDMD-N, caspase-1-P20, mature IL-1β, P-P65 in sham, control and JQ1 group. **(B–D)** Subsequent quantitative analysis of NLRP3, GSDMD-N, P-P65 expression, n=4 mice/group. **(E)** Proteins expression were also reflected by Immunofluorescence intensity in the dorsal horn of the spinal cords at different groups, Bar=50 µm, n=4 mice/group. Data were presented as mean ± standard deviation (SD). (**P < 0.01, ***P < 0.001).

We showed that CFA-induced inflammatory pain was inhibited by the BRD4 inhibitor, JQ1 (**Figure 2B**). The mechanism by which BRD4 inhibition mediated the effect was investigated in more detail. Results showed that the up-regulation of pyroptosis-associated proteins, namely NLRP3, caspase-1-p20, GSDMD-F, GSDMD-N, and mature IL-1β, induced by CFA injection were reduced by JQ1 pre-treatment (**Figures 4A**–**C**). Meanwhile, activation of the NF-κB signaling pathway was significantly reduced by JQ1 pre-treatment through inhibition of p65 phosphorylation (**Figures 4A, D**). Immunofluorescence staining also was used to explore the effects of JQ1 pre-treatment on neuronal pyroptosis. JQ1 pre-treatment alleviated the Immunofluorescence intensity of NLRP3, caspase-1-p20, and GSDMD (**Figure 4E**). These data indicated that JQ1 pre-treatment had a protective effect by inhibiting neuronal pyroptosis.

### 3.5 The BRD4 Inhibitor JQ1 Attenuated Neuronal Pyroptosis *In Vitro*

**Figure 5:**
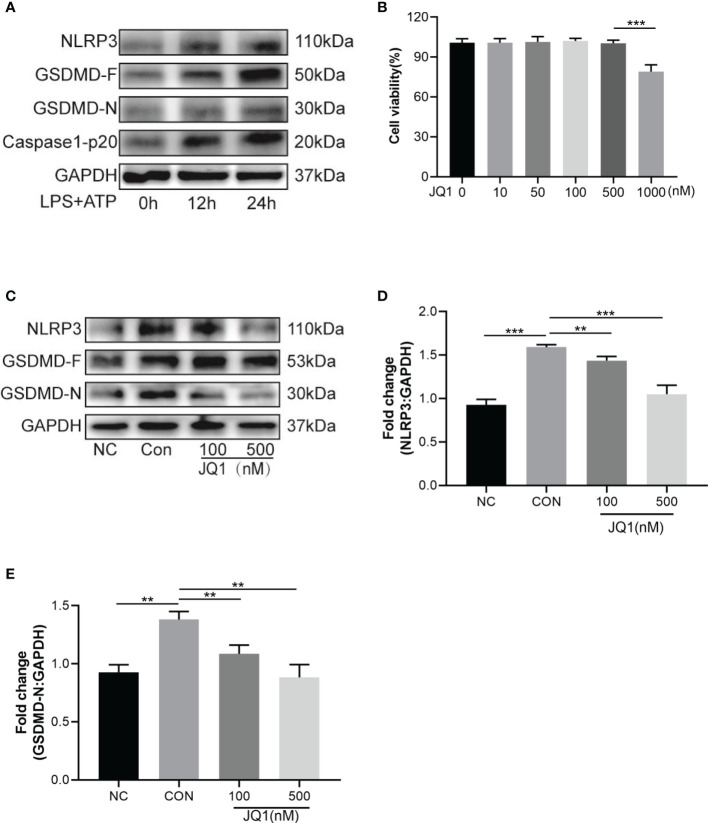
The BRD4 inhibitor JQ1 attenuates neuronal pyroptosis *in vitro*. **(A)** Pyroptosis-related proteins were tested by western blot in HT22 cells stimulated with LPS (1 μg/mL) for 12h, 24h and ATP (5 mM) for 30 min. **(B)** Viability of HT22 cells with different concentrations of JQ1, n = 3 per group. **(C)** Western blot analysis of pyroptosis-related proteins expression in HT22 cells treated by LPS (1 μg/mL) for 24h and ATP (5 mM) for 30 min with or without different concentrations of JQ1. **(D, E)** Semiquantitative analysis of NLRP3 and GSDMD-N. Data are presented as mean ± standard deviation (SD) of three independent experiments. (**P < 0.01, ***P < 0.001).

A newly discovered type of programmed cell death, cell pyroptosis can be induced by LPS and ATP stimulation (22). In this study, HT22 cells were sequentially stimulated with LPS (1 µg/mL) for 12 h or 24 h and 5 mM of ATP for 30 min. The relative levels of pyroptosis-related proteins, namely NLRP3, GSDMD-F, GSDMD-N and caspase-1-p20 were gradually elevated over-time, and reached peaks at 24 h after stimulation (**Figure 5A**). CCK-8 was used to study the effects of JQ1 on the viability of HT22 cells. Results showed that the viability of HT22 cells was inhibited by 1000 nM of JQ1 (**Figure 5B**); therefore 100 nM and 500 nM of JQ1 were chose for subsequent experiments. The up regulation of NLRP3, GSDMD-F, and GSDMD-N proteins induced by LPS and ATP exposure was reversed by pre-treatment of JQ1 (**Figures 5C**–**E**). In summary, data suggested that JQ1, an inhibitor of BRD4, could work against the pyroptosis of HT22 cells *in vitro*.

### 3.6 BRD4 Knockdown Attenuated LPS and ATP-Induced HT22 Cell Pyroptosis

**Figure 6:**
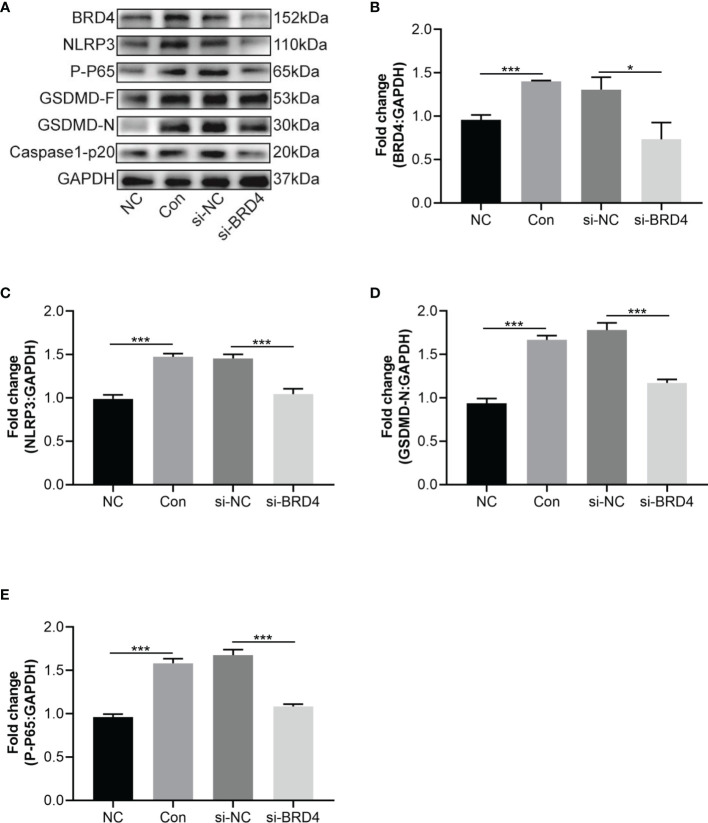
BRD4 knockdown attenuates LPS and ATP-induced HT22 cells pyroptosis. **(A)** Western blot tested NLRP3, GSDMD-F and GSDMD-N in HT22 cells transfected si-RNA targeting BRD4 for 48 h and then stimulated with LPS (1 μg/mL) and ATP (5 mM) for 30 min. **(B–E)** Semiquantitative analysis of NLRP3, GSDMD-F, GSDMD-N and P-P65. Data are presented as mean ± standard deviation (SD) of three independent experiments. (*P < 0.05, ***P < 0.001).

To verify the function of BRD4 in inflammatory pain, we tested the role of pyroptosis in HT22 cells with BRD4 knockdown. First, the effect of BRD4 knockdown was tested by western blot, results showed that the expression of BRD4 in the si-BRD4 group was significantly decreased compared with that of the si-NC group (**Figures 6A, B**). Further, relevant alterations of the phosphorylation p65 induced by LPS stimulation were abolished by BRD4 knockdown, thus inhibiting activation of the NF-κB signaling pathway (**Figures 6A, E**). Notably, NLRP3 inflammasome activation and the cleavage of caspase-1 and GSDMD, which were induced by LPS and ATP stimulation, were markedly alleviated in HT22 cells with BRD4 knockdown (**Figures 6A, C, D**). These results demonstrated that the protective effects of BRD4 knockdown could be attributed to the inhibition of pyroptosis in HT22 cells.

## 4 Discussion

**Figure 7:**
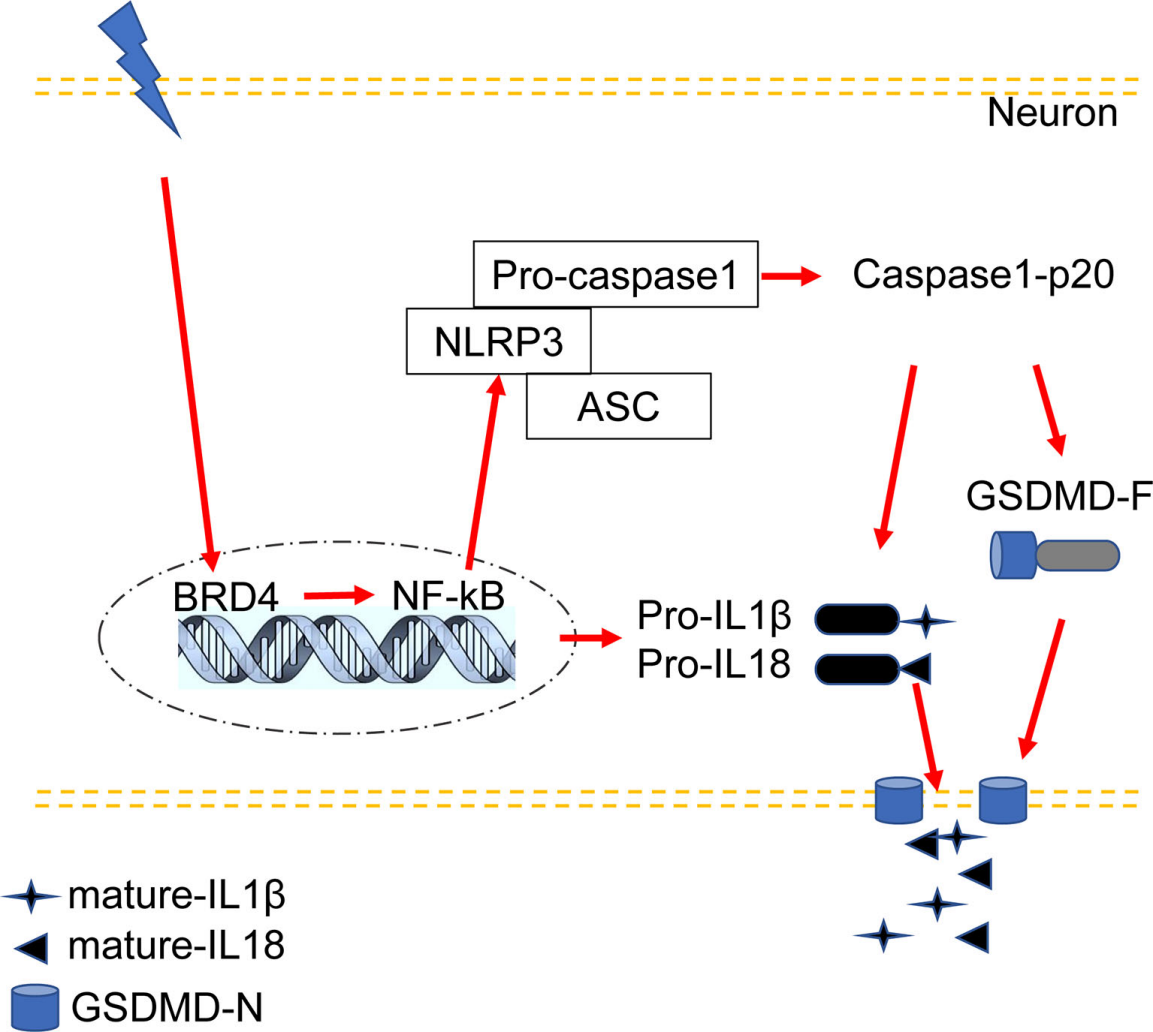
Proposed mechanism: BRD4 regulates NLRP3 inflammasome-induced pyroptosis in CFA-induced inflammatory pain.

This study proposed a new possible mechanism by which BRD4 promotes inflammatory pain hyperalgesia through participation in NLRP3 inflammasome-induced neuronal pyroptosis. The following are highlighted findings of our study: (1) CFA-induced inflammatory pain increased the levels of BRD4 in the spinal dorsal horn, which was mainly colocalized with the marker of neurons. (2) Pyroptosis-related proteins activated by the NLRP3 inflammasome were upregulated in the spinal dorsal horn of mice with CFA-induced inflammatory pain and in HT22 cells exposed to LPS and ATP. The expression levels of NLRP3, GSDMD-F, GSDMD-N, caspase-1-p20, and mature IL-1β were up regulated in a time-dependent manner by the activated NF-κB signaling pathway.Caspase-1-p20 and GSDMD were mainly colocalized with the marker of neurons. (3) BRD4 inhibition by JQ1 could suppress NLRP3 inflammasome-induced neuronal pyroptosis by decreasing the expression levels of the pyroptosis-related proteins both *in vivo* and *in vitro*. (4) BRD4 knockdown produced a similar effect of suppressing GSDMD activation induced neuronal pyroptosis *in vivo*. In summary, BRD4 inhibition was able to alleviate inflammatory pain by ameliorating neuronal pyroptosis activated by the NLRP3 inflammasome (**Figure 7**).

BRD4 has been reported to aggravate mechanical or thermal hyperalgesia of CFA-induced inflammatory pain, and spinal cord injury, nerve injury induced neuropathic pain (5, 8, 11). BRD4 was able to trigger thermal hyperalgesia in CFA-induced inflammatory pain by enhancing the expression of voltage-gated sodium channel 1.7 (8). CFA injection increased the immunofluorescence intensity of BRD4, which was colocalized with a neuron marker in DRG. BRD4 promoted the neuropathic pain induced by HIV glycoprotein 120, and immunofluorescence staining showed that BRD4 was expressed in the neurons of spinal cord dorsal horn (23). Our data also showed that the expression level of BRD4 was consistent with the occurrence of mechanical or thermal hyperalgesia in the spinal cord. Double labeling immunofluorescence staining showed that the marker of neurons, not those of microglia or astrocytes, was the most prominent colocalization with BRD4.

Increasing attention has been given to small molecule inhibitors of BET family members for treating various diseases, including inflammatory diseases and pain (5, 24, 25). JQ1, the most commonly used BRD4 inhibitor, showed a strong anti-inflammatory effect on peripheral and central inflammatory diseases. Macrophage activation increases the expression of various pro-inflammatory cytokines, such as IL-6, IL-1β, and TNF-α, which makes the inflammatory disorders more serious. JQ1 has redressed the imbalance between pro-inflammatory and anti-inflammatory effects by reducing cytokines in the supernatants of LPS-stimulated primary bone marrow-derived macrophages (26). Acute gouty arthritis is a common peripheral inflammatory disease, that results from urate deposition, and no effective treatments are available. BRD4 played a vital role in aggravating the pathological process of acute gouty arthritis, and JQ1 administration has shown great advantages in attenuating joint swelling and synovial inflammation (6). Consistent with the results of that study, our study showed that JQ1 pre-treatment alleviated CFA-induced inflammatory infiltration and paw swelling. JQ1 also showed great advantages in attenuating neuroinflammation. Chronic neuroinflammation is one of the main drivers of Alzheimer’s disease, and JQ1 treatment has reduced the expression levels of the pro-inflammatory modulators IL-6, IL-1β, and TNF-α, suggesting promise in the treatment of neurological disorders (10). Neuroinflammation related to spinal cord injury results in secondary damage. JQ1 has decreased pro-inflammatory cytokine expression and reduced leukocyte recruitment to the injury site (27). Our data implicated that JQ1 was an effective regulator of neuroinflammation, which decreased the levels of mature IL-1β by inhibiting neuronal pyroptosis.

Neuroinflammation contributes to the development of chronic pain (28). Microglia are the main immune cells in the spinal cord that promote the onset of chronic pain (29). Astrocytes also play an important role in maintaining the state of chronic pain (30). Although lots of studies have explored the mechanisms by which acute pain develops into chronic pain, the role of dorsal horn neurons remains elusive in the occurrence of chronic inflammatory pain. Apoptosis of dorsal horn neurons contributes to the development of chronic neuropathic pain (31). Formalin injections have induced selective neuron cell death in the cortex, hippocampus, and hypothalamus in a neonatal rat model of peripheral inflammatory pain (32). In our study, the newly discovered form of programmed cell death, pyroptosis, was induced by CFA-induced inflammatory pain. Immunofluorescence data demonstrated that pyroptosis-related proteins, including GSDMD and caspase-1-p20, were mainly colocalized with the marker of neurons. Neuronal pyroptosis has been reported in central nervous system diseases, such as ischemic stroke (33), and propofol-induced neurotoxicity (34).

Canonical inflammasomes, such as the NLRP3 inflammasome, activate inflammatory programmed cell death. The NLRP3 inflammasome is a sensor of damage-associated molecular patterns, such as LPS and pro-inflammatory modulators (35). Previous evidences has provided insights into the involvement of the NLRP3 inflammasome during inflammatory pain (14, 15). Our data also showed that CFA-induced inflammatory pain increased the NLRP3 inflammasome level in the spinal dorsal horn, as shown by western blot analysis and immunofluorescence staining. Pyroptosis is characterized by the activation of caspase-1, the cleavage of the pore-forming effector, GSDMD, and the release of the pro‐inflammatory cytokines IL‐1β and IL‐18 (36). An activated NLRP3 inflammasome cleaves pro-caspase-1 to caspase-1-p20, which increases mature IL-1β production. GSDMD is cleaved by caspase-1-p20 to become GSDMD-N, causing the formation of membrane pore (37), which increases mature IL-1β and IL-18 secretion.

In the present study, western blot analysis and immunofluorescence staining showed that CFA injection increased the expression levels of GSDMD-N, caspase-1-p20, and mature IL-1β. The NF-κB signaling pathway serves as a mediator of pro-inflammatory cytokines (38). Upon stimulation, the NF-κB subunit p65 will be activated by phosphorylation, promoting activation of the NLRP3 inflammasome signaling (6). Results of our study showed that CFA-induced inflammatory pain elevated the level of p65 phosphorylation in the spinal cord. BRD4 plays a prominent pro-inflammatory role in activating transcription of NF-κB and NF-κB dependent inflammatory genes (39). JQ1 treatment or BRD4 knockdown attenuated the phosphorylation of the NF-κB subunit p65 *in vivo* and *in vitro*, thus inhibiting activation of NLRP3 inflammasome signaling. Taken together, results of our study imply that BRD4 inhibition alleviated NLRP3 inflammasome-mediated pyroptosis *via* the NF‐kB signaling pathway. However, our evidence just indirectly showed that BRD4 inhibition might ameliorate NLRP3 inflammasome-induced pyroptosis. As we known, epigenetic modification plays a vital role in chronic pain. BRD4 plays its biological functions by binding to acetylated histones and transcription factors, regulating multiple pathophysiological activities. BRD4 may up regulate pyroptosis-related proteins by epigenetic modification, such as acetylation or methylation. We are going to investigate more about epigenetic modification regulated by BRD4 in inflammatory pain.

In conclusion, our results provide novel insights into the mechanism by which BRD4 inhibition or knockdown attenuates inflammatory pain. To our knowledge, this study demonstrates for the first time that the benefit of BRD4 inhibition is attributed to alleviation of NLRP3 inflammasome mediated-pyroptosis in CFA-induced inflammatory pain. The finds from this study may provide new ideas for treating chronic inflammatory pain.

## Data Availability Statement

The original contributions presented in the study are included in the article/**Supplementary Material**. Further inquiries can be directed to the corresponding author.

## Ethics Statement

The animal study was reviewed and approved by Institutional Animal Care and Use Ethics Committee of Naval Medical University.

## Author Contributions

HY conceived and designed the study. TH and HW performed animal experiments. TH and XF performed *in vitro* experiments. TH and HW analyzed data and drafted the manuscript. AN, JL, HS, EK, and YL contributed to data analysis, discussion, and revision of the manuscript. All authors read and approved the final manuscript.

## Funding

This work was supported by National Natural Science Foundation of China (81971046, 81901123, 82171220).

## Conflict of Interest

The authors declare that the research was conducted in the absence of any commercial or financial relationships that could be construed as a potential conflict of interest.

## Publisher’s Note

All claims expressed in this article are solely those of the authors and do not necessarily represent those of their affiliated organizations, or those of the publisher, the editors and the reviewers. Any product that may be evaluated in this article, or claim that may be made by its manufacturer, is not guaranteed or endorsed by the publisher.
