# Supplementary material for: BRD4 Inhibition Attenuates Inflammatory Pain by Ameliorating NLRP3 Inflammasome-Induced Pyroptosis
Source: Front Immunol. 2022 Jan 26;13:837977. doi: 10.3389/fimmu.2022.837977 (PMC8826720; doi:10.3389/fimmu.2022.837977)
Supplement: Supplementary file 1 [file DataSheet_1.docx]

Supplementary Material

## Supplementary Figures


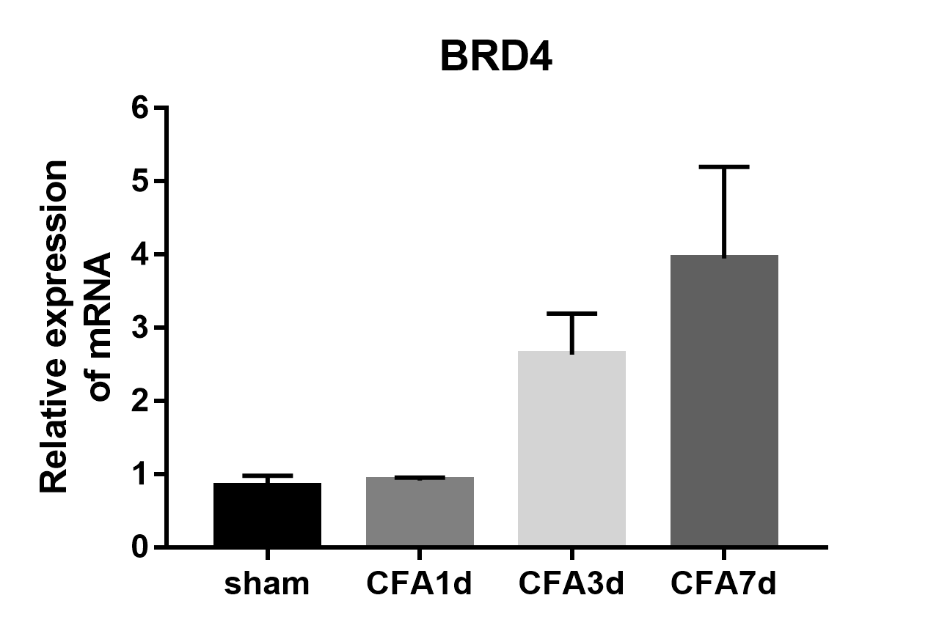


**Supplementary Figure 1.** The mRNA expression of BRD4 was remarkably upregulated, reaching the peak on the 7th day.
